# The American Medical Monthly and New York Reviewer

**Published:** 1860-11

**Authors:** Austin Flint


*The American Medical Monthly and New York Reviewer.* October.—Art. 1.—*Clinical Researches on the Action of Diuretic Remedies*, by Austin Flint, M. D.—The author gives the report of ten cases in which he reports from day to day the treatment, quantity of urine, specific gravity, and amount of solids in twenty-four hours. The acetate of potassa was given in three cases, all of which were cases of sub-acute rheumatism, with the effect of increasing both the quantity and solid constituents of the urine. The nitrate of potassa was given in six cases, viz. four of ascites dependent on cirrhosis, and two of albuminuria from Bright's disease; and in all the cases, save one, the remedy was followed by an immediate increase of the quantity of urine and amount of solids. The exceptional case was one of albuminuria in which vomiting and purging were prominent symptoms, and the remedy appeared to act as a cathartic. Digitalis, squills, and juniper were given in combination in two cases of albuminuria; in one no diuretic effect was produced, but in the other the quantity of urine and the amount of solids were increased in the same proportion. Iodide of potassium was given in one case of albuminuria; the dose was small, and no diuretic effect produced. In one case of subacute rheumatism the wine of colchicum was given, which appeared to increase the amount of solids, while the quantity remained unchanged. The external use of diuretics was employed in three cases, in which a mixture of the tinctures of digitalis, squills, and iodine was applied freely over the abdomen twice daily, accompanied with brisk friction, with apparent diuretic effect. The author concludes that not much reliance can be placed upon the value of diuretics in the treatment of ascites dependent upon cirrhosis, as they augment the solids out of proportion to the increase in quantity, thereby tending to injure rather than benefit the patient; that they may sometimes be usefully employed in the treatment of albuminuria dependent on Bright's disease; that the rational indication in the treatment of subacute rheumatism by diuretics is to increase the solids of the urine, and the acetate of potassa seems to fulfil this indication. He offers to furnish a proper field of study to any competent young man willing to devote a portion of his time in the further pursuit of this subject. Art. 2.—*Lecture on Displacements of the Uterus*, by E. R. Peaslee, M. D. Art. 3.—*Lecture on the Physiology of the Circulation*, by J. C. Dalton, Jr., M. D. Art. 4.—*A new instrument for the local application of anæsthetic and stimulating vapors for deafness, neuralgia*, &c., by H. P. Dewess, M.D., New York. It consists of a delicate retort with nozzel projecting an inch and a half, perforated by a capillary aperture, and a supply-tube rising about an inch above the level of the neck; this being filled with ether is placed in a glass-receiver, filled with moderately warm water, and the nozzle applied to the part affected.—*Amer. Med. Times.*

